# Antiproliferative effects of cadmium sulfide nanoparticles obtained from walnut shells by green synthesis method on SH-SY5Y cell line

**DOI:** 10.1016/j.toxrep.2024.101818

**Published:** 2024-11-19

**Authors:** Yesim Yeni, Hayrunnisa Nadaroglu, M. Sait Ertugrul, Ahmet Hacimuftuoglu, Azize Alayli

**Affiliations:** a Department of Medical Pharmacology, Faculty of Medicine, Malatya Turgut Ozal University, Malatya 44280, Turkey; b Department of Food Technology, Vocational College of Technical Sciences, Ataturk University, Erzurum 25240, Turkey; c Department of Nano-Science and Nano-Engineering, Institute of Science and Technology, Ataturk University, Erzurum 25240, Turkey; d Institute for Cannabis Studies, Ondokuz Mayis University, Samsun, Turkey; e Ataturk University, Faculty of Medicine, Department of Medical Pharmacology, Erzurum 25240, Turkey; f Department of Nursing, Faculty of Health Sciences, Sakarya University of Applied Sciences, Sakarya 54187, Turkey

**Keywords:** Antioxidant, Cadmium sulfide nanoparticles, Green synthesis, Neuroblastoma, SY-SH5Y, Walnut shell extract

## Abstract

Nanoparticles are attracting attention for their potential therapeutic applications, particularly in cancer therapy, underscoring their importance in medicine. Cadmium sulfide nanoparticles, known for their robust catalytic and optical properties, are classified as chalcogenides and show promise for cancer diagnosis and treatment. Neuroblastoma, a common solid tumor in childhood, poses a significant health threat with different outcomes depending on its biological subtype. This study evaluated the antiproliferative effects of cadmium sulfide nanoparticles on the SY-SH5Y cell line. Walnut shell extract and Na_2_S were used to facilitate the synthesis of cadmium sulfide nanoparticles by green synthesis. Characterization of the synthesized cadmium sulfide nanoparticles was performed by Fourier transform infrared spectroscopy, scanning electron microscopy, and x-ray diffraction analyses. The SH-SY5Y cell line was cultured in a standard cell culture medium and then exposed to different cadmium sulfide nanoparticles (10–25–50–75–100 µg/mL) for 24 hours. Cell viability, oxidant, and antioxidant levels were then assessed using a 3-(4,5-dimetiltiyazol-2-il)-2,5-difeniltetrazolyum bromür, total antioxidant, and total oxidant assays. The data showed that applying 100 μg/mL cadmium sulfide nanoparticles resulted in a significant decrease in cancer cell viability of up to 40.96 % (*p*<0.05). The cadmium sulfide nanoparticles had a dose-dependent effect on the SH-SY5Y cell line. Furthermore, cadmium sulfide nanoparticles increased oxidative activity in neuroblastoma cells, which was consistent with the results of the 3-(4,5-dimetiltiyazol-2-il)-2,5-difeniltetrazolyum bromür assay. In conclusion, cadmium sulfide nanoparticles exhibited potent activity against the neuroblastoma cell. This study highlights the antiproliferative efficacy of green-synthesized cadmium sulfide nanoparticles with walnut shell extract on relevant cancer cell lines.

## Highlights

• Green-synthesized CdS NPs from walnut shells exhibit stronger antiproliferative effects on SH-SY5Y cells than cisplatin.
• CdS NPs decrease antioxidant capacity and increase oxidative stress, enhancing their cytotoxic impact on SH-SY5Y cells.
• Walnut shell-based CdS NPs offer a sustainable and effective alternative for targeting SH-SY5Y cells with improved efficacy.

## Graphical Abstract

**Figure:**
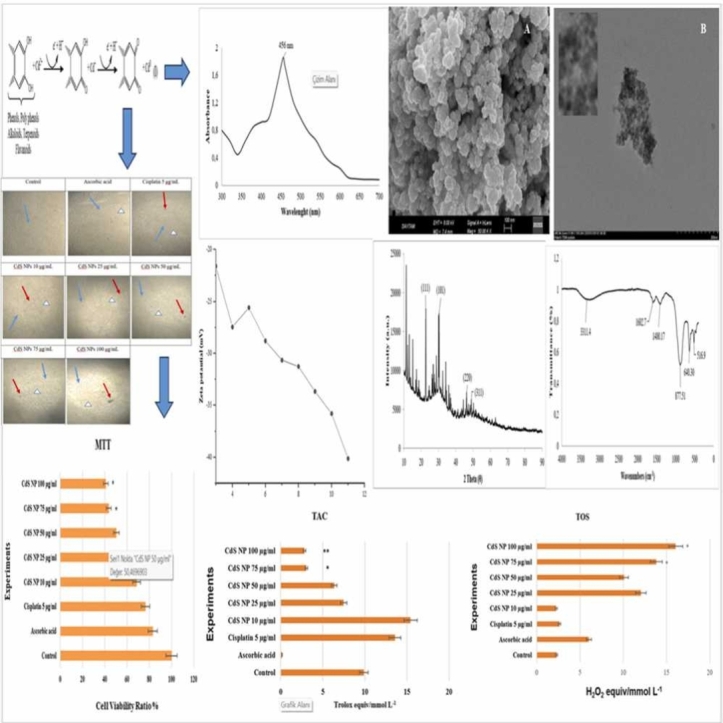

## 1 Introduction

Neuroblastoma (NB) is the most common solid tumor, accounting for approximately 15 % of cancer-related deaths in children [1], [2]. These undifferentiated cells can spontaneously return to the malignant type or develop into benign ganglia. The incidence of NB is 1 in every 100,000 children, especially those under 5 years of age [3]. Only 40 % of these events are diagnosed in children under one year of age [4]. High-risk patients respond inadequately to treatments, and overall survival for these patients is between 40 % and 50 %. The biology of the tumor influences this survival rate, the site of origin, and the age of the disease [5], [6]. Clinically, efforts to improve treatment and diagnosis are crucial, and new agents or adjuvants are needed to reduce the dose of antitumor drugs [7]. In a study by Sonawane et al., they determined that protein-coated CdS nanoparticles act as inhibitors of Tau aggregation, whose accumulation in the brain is the cause of neuroblastoma. It was also determined that the inhibition of tau fibrillization was inhibited by protein-coated Fe_3_O_4_ NPs [8].

Current anticancer drugs are expensive, and most patients respond to chemotherapy first and then develop resistance [9], [10], [11]. Therefore, it is important to develop a biocompatible and economical method for curing cancer. Metallic nanoparticles (cadmium, silver, and copper), which are among the therapeutic approaches, have recently attracted increasing amounts of attention but have shown toxic effects in vitro [12], [13]. In this context, cadmium nanoparticles (Cd NPs) have been shown to induce apoptosis by inhibiting DNA repair and causing free radical-induced DNA damage, mitochondrial damage, and intracellular calcium signal degradation [14], [15].

Cd damages the liver when it is bulky and causes less damage when it is applied with CdS or Na_2_S_4_ in addition to Cd [16]. In a study, they determined that in the investigation of toxicity in the biological system of quantum dot (QD) formulations containing Cd NPs, the toxicity that occurs in cell culture is not due to only one factor but rather to an element combination of the particle formulations. Different cytotoxicity trends were found for all prepared QD formulations tested on gastric adenocarcinoma (BGC-823) and neuroblastoma (SH-SY5Y) cell lines used in toxicity assessments [17].

Recently, biological/green synthesis of NPs has been preferred as an alternative to physical and chemical methods. Therefore, the waste products of plant extracts are non-toxic and easy to dispose of. Due to such advantages, it meets the need for less toxic and more effective treatment options [16]. In addition, green synthesized NPs play important roles in areas such as diagnosis, drug therapy, anticancer, antimicrobial and anti-inflammatory activities, and treatment of diseases [17], [18]. Based on this information, in the present study, CdS NPs were synthesized by the green synthesis method using walnut shell extract containing the reducing agent Na_2_S. We also investigated the antiproliferative effect of CdS NPs on the SH-SY5Y cell line. An important goal of our study was to extend the benefits of CdS NPs for the treatment of NB**.**

## 2 Materials & methods

### 2.1 Materials

Cisplatin from Kocak Pharma Ltd. (Tekirdag, Turkey) was supplied. Fetal bovine serum (FBS), Dulbecco’s modified Eagle’s medium/F12 (DMEM/F12), phosphate buffer solution (PBS), L glutamine, trypsin-EDTA, and antibiotic antimitotic solution (100×) were acquired from Sigma Aldrich (St. Louis, MO, USA).

### 2.2 Preparation of plant extract

For the production of CdS NPs, the walnut shell used as a reducing agent in green synthesis was collected from a walnut orchard in Erzurum Province and evaluated by taxonomists. After the walnut shells were washed, 25 g of the walnut shells were removed and crushed with a chopper. The mixture was then filtered and centrifuged at 5000×g for 10 min, and the resulting supernatant was used for green synthesis [13], [19].

The walnut extract was added to a 0.1 M Cd(NO_3_)_2_ solution at a volume ratio of 1:5 and stirred for 15 min at 300 rpm at room temperature. Then, 100 mL of 0.1 M Na_2_S solution was added dropwise to the medium with the help of a dropping funnel. To complete the reaction, the reaction was continued at room temperature for 12 h. The obtained CdS NPs were first washed with distilled water and then with ethyl alcohol and then dried in an oven at 40 °C under vacuum [18], [20].

### 2.3 Green synthesis and characterization of CdS NPs

CdS NPs were first synthesized using walnut shell extract, Cd(NO_3_)_2_, and NaS. The surface topography of the CdS NPs was determined by scanning electron microscopy (SEM) using a Metek Apollo prime with an active area of 10 mm^2^ (Microscope Inspect S50). Then, X-ray diffraction (XRD) analysis was performed to determine the crystallinity of the CdS NPs using a Panalitic Empyrean instrument equipped with a Ni filter and Cu Kα radiation source (λ = 0.1542 nm) in the range of 10–80° at a scan rate of 4° min^−1^. Fourier transform infrared spectroscopy (FTIR) analysis of the CdS NPs was performed using a Vertex 80 Model FTIR Frontier spectrophotometer via the attenuated total reflection (ATR) technique in the 4000–400 cm^−1^ region [21], [22], [23].

### 2.4 Cell cultures dose studies

The SH-SY5Y cell lines were obtained from the Department of Medical Pharmacology Department of Ataturk University (Erzurum, Turkey). Briefly, after the cells were centrifuged at 1200 rpm for 5 min, a suspension with the prepared medium (DMEM/F12 medium, 1 % antibiotic, and 15 % FBS) was obtained at a density of 1×10^5^ cells/mL. Then, 400 µL of cell suspension was added to each well. The cells were transferred to 24-well plates and kept in an incubator (95 % humidity, 37°C, and 5 % CO_2_) [24].

### 2.5 Administration of CdS NPs

CdS NPs were dissolved in 1 % ascorbic acid. After the cells in the 24-well plates reached 85 % confluence, CdS NPs (10, 25, 50, 75, and 100 μg/mL) in the prepared medium (DMEM/F12 medium, 1 % antibiotic, and 15 % FBS) were added to the wells, and the plates were incubated for 24 h in 5 % CO_2_ at 95 % moisture and 37 °C. For this purpose, the experimental groups were divided into the following 8 groups: Control, Ascorbic acid (0.05 %), Cisplatin (5 μg/mL), and CdS NPs (10, 25, 50, 75, and 100 μg/mL).

### 2.6 MTT assay

The MTT protocol was applied after 24 hours of incubation with the test substances. The medium in the wells was removed by the aspiration method. Then, 180 µL of medium and 20 µL of MTT solution were added to the wells. The cells were incubated with MTT solution for 4 h. After incubation, the MTT solution in the wells was removed by the aspiration method, and the formazan crystals formed were dissolved in 100 µL of DMSO. The change in absorbance was monitored at a wavelength of 570 nm (Multiskan™ GO Microplate Spectrophotometer reader).

### 2.7 Total antioxidant capacity (TAC)

The kit consists of 4 components: Reagent 1, Reagent 2, Standard 1 and Standard 2. Standard 2 in the kit was used for Standard 2, while distilled water was used for Standard 1. Briefly, 500 µL of Reagent 1 was added to the wells containing 30 µl of sample, and the first absorbance was read at 660 nm (Multiskan™ Microplate Spectrophotometer reader). Then, 75 µL of Reagent 2 was added to the wells and incubated at room temperature for 10 min. At the end of the waiting duration, the secondary absorbance value was read at 660 nm. The results obtained were expressed as Trolox equivalents/mmol/L.

### 2.8 Total oxidant status (TOS)

The kit consists of 4 components: Reagent 1, Reagent 2, Standard 1 and Standard 2. Standard 2 in the kit was used for Standard 2, while distilled water was used for Standard 1. Briefly, 500 µL of Reagent 1 was added to the wells containing 75 µL of sample, and the initial absorbance was read at 530 nm (Multiskan™ Microplate Spectrophotometer reader). Then, 25 µL of Reagent 2 was added to the wells and incubated at room temperature for 10 min. At the end of the waiting duration, the secondary absorbance value was read at 530 nm. The results obtained were determined in units of H_2_O_2_ equiv/mmol. L^−1^ [18].

### 2.9 Morphological determination

All groups’ images were taken after 24 hours of exposure. For this purpose, all images were taken at a 20× magnification. Morphological determination was performed using a Leica microscope (USA).

### 2.10 Statistical analyses

The results are expressed as the mean ± standard deviation. The analyses were performed by one-way variance (ANOVA) and Tukey’s honestly significant difference (HSD) test using SPSS 22.0 software. *p<0.05* was considered a statistically significant difference for all tests.

## 3 Results

In this study, CdS NPs were characterized using FTIR, SEM, and XRD, and the antitumor effect of CdS NPs on the SH-SY5Y cell line was evaluated. These cells were exposed to CdS NPs (10, 25, 50, 75, and 100 μg/mL) for 24 hours. MTT, TOS, and TAC tests were performed after the indicated exposure times.

### 3.1 Mechanism of the green-synthesis of CdS

**Fig. 1:**
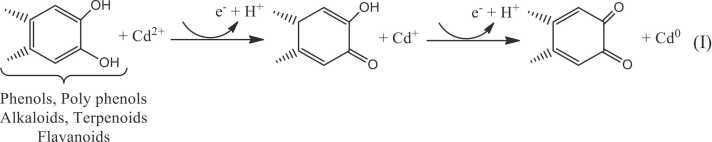
The synthesis mechanism of CdS NPs.

Phytochemicals found in plant extracts can reduce metal ions to nanoparticles in a shorter time. Plant extracts play a role in NP synthesis reactions, not only by having a reducing capacity but also as a stabilizing agent. The flavonoids, terpenes, alkaloids, phenols, polyphenols, sugars, ketones, aldehydes, carboxylic acids, and amides, in the walnut shell extract used in our research are responsible for bioreduction during the synthesis of CdS NPs. The synthesis mechanism of CdS NPs using phenolic groups is given in reaction (I) (Fig. 1). In addition, FTIR analysis of the obtained CdS NPs confirmed that bioagents play a role in the synthesis [25].

### 3.2 Characterization of the synthesized CdS NPs

#### 3.2.1 UV—visible absorption spectroscopy

**Fig. 2:**
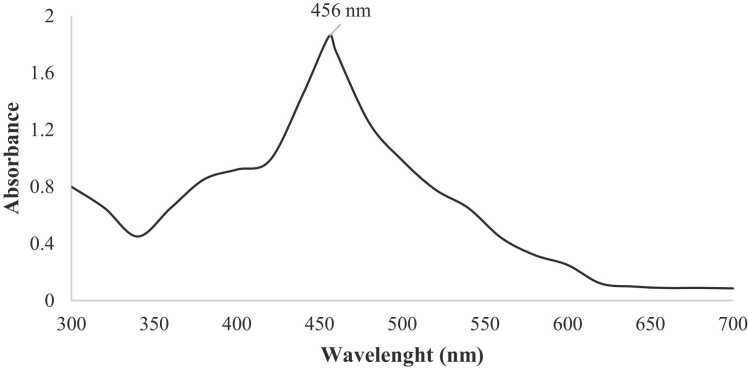
UV- Vis spectra of CdS NPs synthesized by walnut shell using 0.1 M Cd(NO_3_)_2_, 0.1 M Na_2_S.

The optical properties of the CdS NPs were examined by UV—Vis spectroscopy. For this purpose, wavelength scanning of CdS NPs formed in reaction media surface plasmon resonance bands plays a vital role in determining their size, shape, and morphology. Fig. 2 shows the UV—visible spectrum of the nanoparticles obtained from the walnut shell extract. The synthesized CdS NPs exhibited a strong plasmon resonance band at 456 nm. UV—Vis spectroscopy usually shows the presence of green CdS NPs. In particular, the absorbance between 400 nm and 450 nm has often been used as an indicator of Cd^2+^ reduction [26].

#### 3.2.2 Surface characterization of CdS NPs

**Fig. 3:**
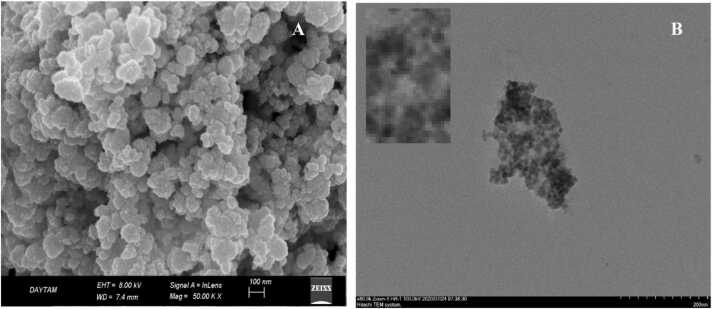
SEM image (A) and TEM image (B) of CdS NPs.

The chemical and mineralogical compositions of the synthesized green CdS NPs were determined by SEM, which was used to examine the surface of the adsorbent. The images of the CdS NPs were magnified 10,000 times by Zeiss, with an active area of 10 mm^2^ (Fig. 3A). TEM (transmission electron microscopy) images of the CdS NPs synthesized using a Hitachi HighTech HT7700 instrument were obtained (Fig. 3B).

SEM analysis was used to evaluate the composition and purity of the CdS NPs obtained by the green synthesis method. As shown in Fig. 3A, the CdS samples were determined to have almost cubic particles, and the overall morphology of the particles was almost homogeneous. The CdS NPs had a nearly spherical arrangement on the smooth surface and formed parallel layers with diameters in the range of 5–35±1.0 nm and an average diameter of 20±1.5 nm.

The CdS NPs obtained by the green synthesis method were also characterized by TEM (Fig. 3B). The electron micrograph shows that the NPs are homogeneously distributed in an amorphous matrix of low crystallinity. A digital zoom of the TEM image shows that these QDs are normal polyhedrons (Fig. 3B), similar to hexagonal NP models. From the TEM image, it was determined that the CdS NPs were smaller than 100 nm and were in the 5–35 nm size range, which supported the results of the SEM analysis.

**Fig. 4:**
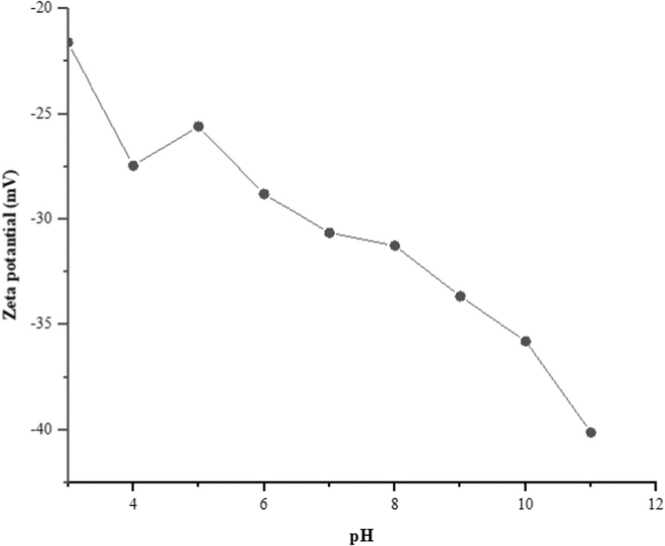
Effect of pH on zeta potential of CdS NPs.

Fig. 4 shows the effect of different pH values on the zeta potentials of CdS NPs in pure water. In colloidal systems, it is necessary to have a high zeta potential to prevent agglomeration. When the findings were examined, it was determined that CdS NPs reached a high value of 40.12 mV at pH 11, where it was quite stable above pH 6.0. Although the zeta potential shows the total charge obtained in a particular environment, the negative zeta potential values indicate that the CdS NPs formed a double layer. Saxena et al. [27], while obtaining unstable results at pH 3.19, determined a high zeta potential representing a stable structure above pH 10.32.

#### 3.2.3 XRD of CdS NPs

**Fig. 5:**
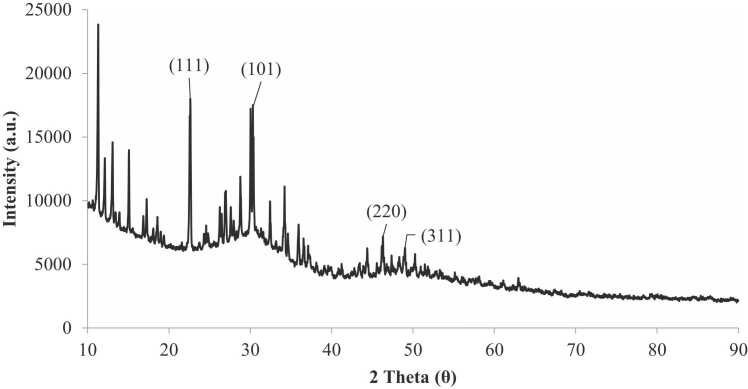
X-ray diffraction pattern of CdS NPs.

The XRD pattern of the CdS NPs produced from the catalyst and their crystallographic analysis are shown in Fig. 5. The characteristic peaks in the XRD spectrum at 2θ = 22.62°, 30.06°, 46.28° and 48.86° that can be indexed to the (111), (101), (220), and (311) planes, respectively. Similar observations were reported by Zhu and Tong, who synthesized CdS using cadmium chloride and thiourea [28].

#### 3.2.4 FTIR analysis

**Fig. 6:**
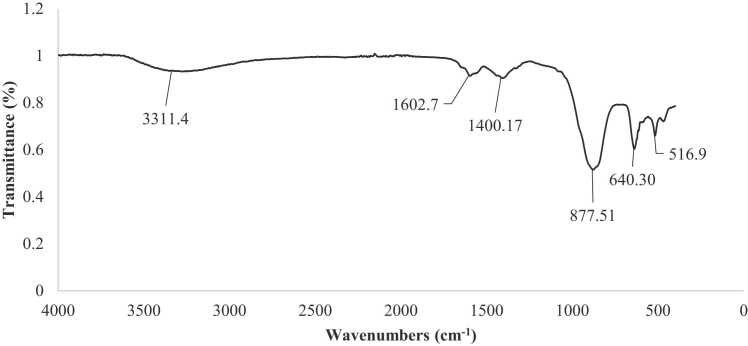
FTIR spectrum of CdS NPs.

FTIR was used to identify the possible biomolecules responsible for the reduction of CdS NPs via green synthesis. Fig. 6 shows the FTIR spectrum of CdS NPs synthesized from walnut shell plant extract and Na_2_S. The spectra show bands at 3311.4, 1602.7, 1400.17, 877.51, and 640.30 cm^−1^. The sharp band at 1602.7 cm^−1^ represents the C=O vibrations typical of the structure of flavonoids that can be found in walnut shells. The absorption band at 1400.2 cm^−1^ is related to the -C–H bending vibrations of the aromatic amine groups in the flavonoid structure. The peak observed at approximately 877.51 cm^−1^ indicates the presence of CdS NPs and is characteristic of the C-S bond structure. The observed peak at 640.30 cm^−1^ belongs to the -CH_2_ group in the aliphatic chain structure. The FTIR spectrum confirmed the presence of bioactive compounds in the walnut shell plant. Similar observations were also reported by Jameel et al., who synthesized CdS NPs using Nopal Cactus fruit extract by green synthesis method [29].

### 3.3 Results of the antitumor effects of CdS NPs and cisplatin

In this study, the antitumor effects of CdS NPs and cisplatin were evaluated. For this purpose, cisplatin was kept constant, and the effects of different concentrations of CdS NPs were examined [30].

#### 3.3.1 MTT assay

**Fig. 7:**
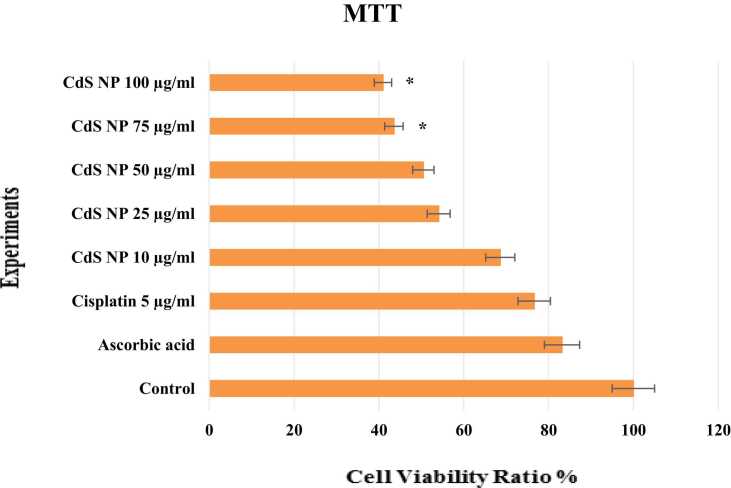
Viability rates for SH-SY5Y cancer cells - MTT assay. *p <0.05,^**^p< 0.001.

The survival rate of cancer cells after 24 h of drug exposure was calculated by using the MTT assay (Fig. 7). Accordingly, all doses administered after 24 h of exposure were found to have lower cell viability than cisplatin, the positive control. Notably, the effectiveness of CdS NPs increased with increasing doses, and the cytotoxic effect on the cancer cell line became more pronounced. The highest anticancer efficacy dose of CdS NPs was determined to be 100 µg/mL. The percentage of cancer cells at this dose decreased by up to 40.96 %. According to the data obtained, all doses of CdS NPs are effective, but the most effective results are in the range of 75–100 µg/mL (43.53–40.96 %, respectively). The 75 and 100 μg/mL CdS NP groups were significantly different from the positive control group *(p<0.05*).

#### 3.3.2 TAC assay

**Fig. 8:**
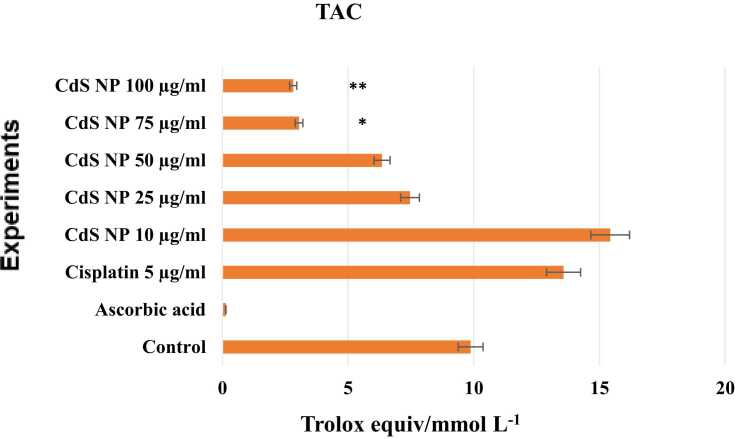
Total antioxidant capacity test values read spectrophotometrically at 660 nm in cell culture fluid. *p<0.05,^**^p< 0.001.

A TAC kit was applied to the medium taken from the wells 24 hours after drug administration. We evaluated the TAC test based on Trolox equiv/mmol/L (Fig. 8). The TAC levels of the control and positive control groups were 9.8 and 13.5 %, respectively. Compared to the control and positive control, high doses of CdS NPs had very low antioxidant activity. Compared with those in the control and positive control groups, the antioxidant levels in the groups with concentrations other than 10 µg/mL CdS NPs (7.4, 6.3, 3, and 2.8 ng/mL) decreased. A significant difference was observed between the groups treated with 100 μg/mL CdS NPs *(P<0.001)* or 75 μg/mL CdS NPs *(p<0.05)* and the positive control group. The antioxidant activity of the CdS NPs at lower doses was greater than that at higher doses.

#### 3.3.3 TOS assay

**Fig. 9:**
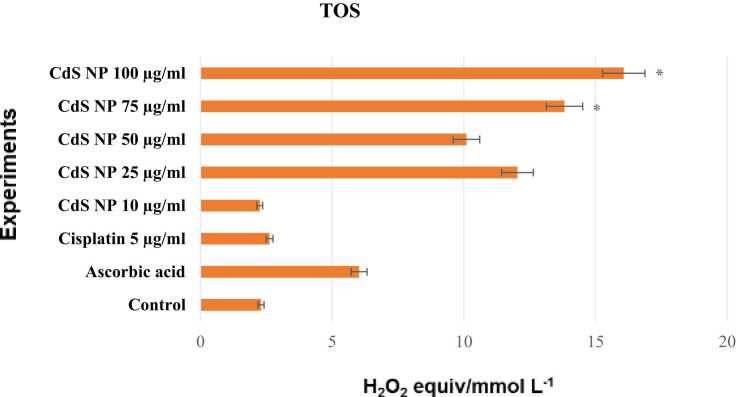
Total oxidant status test values read spectrophotometrically at 530 nm in cell culture fluid. *p<0.05,^**^p< 0.001.

We evaluated the TOS test based on H_2_O_2_ equiv/mmol L^−1^ (Fig. 9). The TOS levels of the control and positive control groups based on H_2_O_2_ equiv/mmol/L were 2.3 and 2.6, respectively. In the groups, oxidant levels were increased compared to those in the control group, except for the 10 μg/mL CdS NP group. Accordingly, the TOS values at doses ranging from 75 to 100 µg/mL were 13.82 and 16.07, respectively. In addition, the oxidant level was greater in the 100 μg/mL CdS NP group. A statistically significant difference (*p<0.05*) was observed between the 75 and 100 μg/mL CdS NP groups. Furthermore, there was no statistically significant difference between the 10 μg/mL CdS NP group and the positive control group (*p>0.05*). Almost all of the TOS data indicate that the oxidant activity increases in a dose-dependent manner.

#### 3.3.4 Morphological determination

**Fig. 10:**
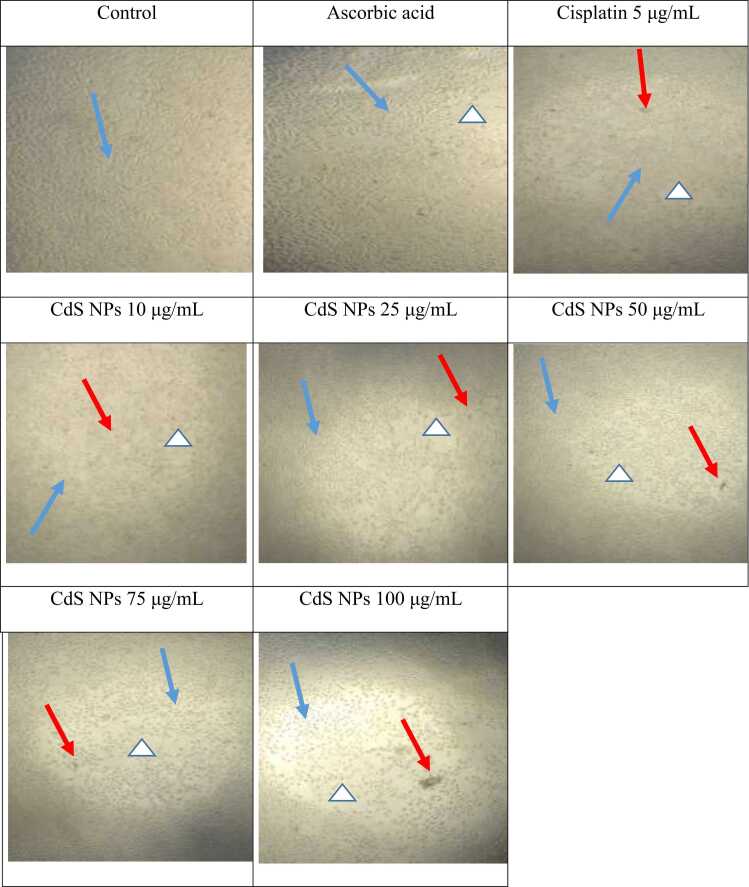
Microscopic view of each group after 24-hour treatment (40×). Blue arrow: Live cells, Red arrow: Died cells, Triangle: Empty space.

After 24 hours of treatment, images of all groups were taken at 40× magnification (Fig. 10). The control group had the largest number of live cells, whereas the 75 μg/mL CdS NP and 100 µg/mL CdS NP groups had the least number of live cells. These results are consistent with the MTT results.

## 4 Discussion

NB is biologically heterogeneous; the low-risk model can be withdrawn automatically, but as it grows, the high-risk model is constantly expanding and can be fatal [1], [7], [31], [32]. This type of cancer, which is very rare in adults, is common in children [1], [2], [7]. It is difficult to detect on time, as symptoms are rare in adults until the disease has metastasized [7]. In this regard, green-synthesized CdS NPs, which are thought to be promising for use in children, may not be effective in adult patients.

Currently, nanoparticles are widely used in scientific studies and production [33]. Biological safety assessments of nanotechnology are necessary not only for medical science but also for the sustainable development of the environment and human health [34], [35], [36]. Therefore, there are studies on the toxicity and environmental evaluation of nanoparticles [36], [37] Semiconductive CdS NPs are widely used, especially because of their high chemical activity and large specific surface area. However, it remains a mystery whether CdS NPs have a detrimental effect on human health [38].

According to previous studies, exposure to CdS NPs can cause serious damage to various parts of the body, as it can cause toxicity and health problems [39], [40]. In a study by Hossain and Mukherjee, CdS NPs increased reactive oxygen species (ROS) levels and induced oxidative stress in HeLa cells [41]. Moreover, some studies have confirmed that NPs can damage cells through oxidative stress caused by ROS [42]. Another study of A549 cell survival after treatment with CdS NPs revealed that CdS NPs increased cellular mortality [38].

Wang L et al. studied the cytotoxic effects of CdS NPs (25 μM, 50 μM, and 100 μM) on a rat glioma cell line at different concentrations of ionic liquids. This study showed an increased cytotoxic effect and increased NP concentrations with CdS NPs biosynthesized in the presence of ionic liquids [43]. In addition, treatment of MDA-MB-468 and MCF-7 cells with cadmium tellurium quantum dots inhibited cell growth in a concentration-dependent manner [44]. As shown in Fig. 7, our MTT results are by those of previous studies. According to the MTT results, 100 µg/mL CdS NPs had a cytotoxic effect on the NB cancer cell line by reducing viability by 40.96 %. This rate was much greater than the effectiveness of cisplatin, a drug that is included in the standard treatment of the NB cancer line. CdS NPs exhibited more potent toxicity to the SH-SY5Y cell line than did cisplatin. There are no studies in the literature investigating the effect of the green-synthesized CdS NPs with walnut shell extract in the SH-SY5Y cell line.

Compared with those of the control, the antioxidant effectiveness of the CdS NPs decreased, especially at doses of 75 and 100 µg/mL, where the anticancer efficacy was greatest. The increased antioxidant activity in NB cells treated with cisplatin is consistent with the data obtained from the literature [41]. It is not known whether nanostructures that act on the NB cancer cell line and cisplatin differ in their antioxidant activity. However, in studies with different nanoparticles, it has been noted that it does not change the level of antioxidant activity (TAC) in cancer lines [42]. In addition, the highest TOS levels were found at 75 and 100 µg/mL, which are the two most effective doses of CdS NPs in terms of anticancer activity. This study revealed that the increase in the oxidant concentration and the decrease in the antioxidant activity were due to the increased antioxidant activity in the treatment groups.

The results showed that the CdS NPs applied to the SH-SY5Y cell line were relatively more effective than cisplatin. The results obtained in a relatively dose-dependent manner showed that the analyses performed were interrelated. Accordingly, a 100 µg/mL CdS NP dose was determined to be an acceptable new treatment approach for highly aggressive cancer-type NB.

## 5 Conclusion

The results showed that CdS NPs synthesized and characterized using a green synthesis method had a more pronounced effect on the SH-SY5Y cell line than did the drug cisplatin. This dose-dependent effect is likely attributed to oxidative stress induced by the increased levels of reactive oxygen species within the cancer cells. Our results demonstrate that CdS NPs inhibit the progression of the SH-SY5Y cell line by inhibiting cell proliferation, reducing viability, and suppressing cancer cell invasion. CdS NPs have potential as novel anticancer agents for the treatment of NB patients or as an adjunct to mitigate the side effects of chemotherapy. Therefore, further research is warranted to determine a safe dose of CdS NPs that maximizes their therapeutic efficacy.

## Scientific responsibility statement

The authors declare that they are responsible for the article’s scientific content, including study design, data collection, analysis and interpretation, writing, some of the main lines, or all of the preparation and scientific review of the contents and approval of the final version of the article.

## Animal and human rights statement

All procedures performed in this study were in accordance with the ethical standards of the institutional and/or national research committee and with the 1964 Helsinki Declaration and its later amendments or comparable ethical standards. No animal or human studies were carried out by the authors of this article.

## Funding

There are currently no funding sources.

## CRediT authorship contribution statement

**Hayrunnisa Nadaroglu:** Writing – original draft, Formal analysis. **M. Sait Ertugrul:** Writing – original draft, Formal analysis. **Yesim Yeni:** Writing – original draft, Formal analysis. **Ahmet Hacimuftuoglu:** Writing – original draft, Formal analysis. **Azize Alayli:** Writing – original draft, Formal analysis.

## Declaration of Competing Interest

The authors declare that they have no known competing financial interests or personal relationships that could have appeared to influence the work reported in this paper.

## Data Availability

Data will be made available on request.
